# Greener Route for Synthesis of aryl and alkyl-14H-dibenzo [a.j] xanthenes using Graphene Oxide-Copper Ferrite Nanocomposite as a Recyclable Heterogeneous Catalyst

**DOI:** 10.1038/srep42975

**Published:** 2017-02-24

**Authors:** Aniket Kumar, Lipeeka Rout, Lakkoji Satish Kumar Achary, Rajendra. S. Dhaka, Priyabrat Dash

**Affiliations:** 1 Department of Chemistry, National Institute of Technology, Rourkela, Odisha, 769008, India; 2 Novel Materials and Interface Physics Laboratory, Department of Physics, Indian Institute of Technology Delhi, Hauz Khas, New Delhi, 110016, India

## Abstract

A facile, efficient and environmentally-friendly protocol for the synthesis of xanthenes by graphene oxide based nanocomposite (GO-CuFe_2_O_4_) has been developed by one-pot condensation route. The nanocomposite was designed by decorating copper ferrite nanoparticles on graphene oxide (GO) surface via a solution combustion route without the use of template. The as-synthesized GO-CuFe_2_O_4_ composite was comprehensively characterized by XRD, FTIR, Raman, SEM, EDX, HRTEM with EDS mapping, XPS, N_2_ adsorption-desorption and ICP-OES techniques. This nanocomposite was then used in an operationally simple, cost effective, efficient and environmentally benign synthesis of 14H-dibenzo xanthene under solvent free condition. The present approach offers several advantages such as short reaction times, high yields, easy purification, a cleaner reaction, ease of recovery and reusability of the catalyst by a magnetic field. Based upon various controlled reaction results, a possible mechanism for xanthene synthesis over GO-CuFe_2_O_4_ catalyst was proposed. The superior catalytic activity of the GO-CuFe_2_O_4_ nanocomposite can be attributed to the synergistic interaction between GO and CuFe_2_O_4_ nanoparticles, high surface area and presence of small sized CuFe_2_O_4_ NPs. This versatile GO-CuFe_2_O_4_ nanocomposite synthesized via combustion method holds great promise for applications in wide range of industrially important catalytic reactions.

Xanthenes are important heterocycles with a variety of applications in the field of pharmaceutical chemistry^1^. Notable pharmaceutical properties of xanthene derivatives are antibacterial^2^, analgesic, antiviral^3^, anti-inflammatory^4^, antimalarial^5^ and anticancer^6^. These compounds have found wide use in dyes^7^, laser technologies and as pH-sensitive fluorescent materials^8^. Because of their usefulness, research on the catalytic preparation of xanthenes has attracted great attention. Mainly, condensation of aldehyde and 2-naphthol is the usual procedure for library synthesis of xanthenes, and its structural variants. A wide variety of catalysts have been reported in literatures for the synthesis of xanthenes such as *p*TSA^9^, sulfamic acid^10^, molecular iodine^11^, tungsten heteropoly acid, silica sulphuric acid^12^, NaHSO_4_–SiO_2_^13^, TiO_2_–SO_4_^−2 ^14, amberlyst-15^15^, wet cyanuric chloride^16^, K_5_CoW_12_O_40_.3H_2_O, acyclic acidic ionic liquids^17^, cellulose-sulphuric acid^18^, boric acid^19^ and Yb(OTf)_3_^20^. However, these catalysts suffer from one or more disadvantages, such as long reaction times, unsatisfactory yields, harsh reaction conditions, time taking work-ups, high cost, toxic solvent and difficulty in separation, justifying considerable scope for development of a noble catalyst for the synthesis of xanthenes via facile, energy efficient, easy separable and environmentally benign process.

Nanocatalysis, involving nanoparticles as catalyst has shown tremendous applications for a variety of organic transformations. Upto now, many investigations have been done on nanocatalysis, but there still remains the challenge of recovery of nanocatalyst from the reaction mixture. For this reason, magnetic nanoparticles have recently emerged as a useful group of nanocatalyst. The separation of magnetic nanoparticles is found to be simple and economical which diminishes the loss of catalyst, resulting in enhanced reusability. In addition, they exhibit high catalytic activity due to their large surface area and have relatively low preparation costs and toxicity. All these properties make them desirable and promising catalysts^21^,^22^,^23^,^24^ for industrial applications.

Among various magnetic nanoparticles, the copper ferrite, CuFe_2_O_4_ with a spinel structure, has been widely used in sensors, electronics and catalysis owing to its unique advantages such as environmental compatibility, moisture insensitive, high dispersion, high reactivity, low cost and easy separation by an external magnet. In catalysis, they are found to be a promising material for a variety of catalytic applications. For example, Amini *et al*. investigated the low temperature CO oxidation over mesoporous CuFe_2_O_4_ nanopowders synthesized by a novel sol–gel method^25^. Parella *et al*. explored the catalytic application of CuFe_2_O_4_ nanoparticles for the Friedel–Crafts acylation^26^. Feng *et al*. investigated the catalytic activity of CuFe_2_O_4_ nanoparticles for the reduction of 4-nitrophenol to 4-aminophenol with an excess amount of NaBH_4_^27^. To further improve the application efficiency of nanoparticles, various strategies have been employed over the years. One of the effective strategies is depositing nanoparticles on various carbon supports. These carbon hybrids are found to be highly active and selective catalyst as the result of the synergistic combination of both nanoparticle and carbon supports.

Graphene oxide, a two-dimensional sheet of sp^2^ hybridized carbon has received increasing attention as it possesses similar properties to that of graphene. Because of its high surface area, mechanical and electrical properties and thermal stability it has been used as a significant supporting material and has been found as a promising material for fuel cells^28^, sensors^29^, solar cells^30^, lithium batteries^31^ and organic synthesis^32^. In the catalysis front, owing to its large specific surface area, high chemical stability, good adsorption capacity, highly active and highly selective GO-based nanocatalysts have been designed by decorating GO surface with nanoparticles. The combination of the NPs and the graphene oxide sheets affords the composite better performance due to the synergistic interaction between the NPs and the graphene oxide sheets. These GO-based nanocatalyst mimic both homogeneous (high surface area and easily accessible) as well as heterogeneous (stable and easy to handle) catalyst systems^33^. In addition to this, the presence of polar oxygen-containing functional groups, such as hydroxyl, epoxy, and carboxyl groups around GO prevents metal oxide nanoparticles from aggregation and leaching. Various metal oxides nanoparticles such as Fe_3_O_4_^34^, CoFe_2_O_4_^35^, ZnFe_2_O_4_^36^, TiO_2_^37^, SnO_2_^38^ and ZnO^39^ were loaded on graphene oxide (GO) sheets, generating highly active and selective catalysts. Recently, our group designed graphene oxide–SnO_2_ nanocomposite (GO–SnO_2_) and used it as an efficient catalyst for the β-enaminones synthesis^40^. Considering the benefits associated with both CuFe_2_O_4_ nanoparticles and GO, the combination of GO with CuFe_2_O_4_ would form a potential catalytic material and more recently, few studies have corroborated this fact^27^,^41^. However, to our best knowledge, there has been no report yet on the use of graphene oxide-copper ferrite (GO-CuFe_2_O_4_) magnetic nanocomposite as a catalyst for biologically important xanthene derivatives syntheses.

The methods to synthesize GO-based nanocomposites are diverse, such as hydrothermal^27^, solvothermal^42^, and co-precipitation method^43^. However above mentioned methods had some disadvantages such as time consuming, expensive, pollution causing and low yields. One method which has attracted a great deal of research in generating various metal oxide nanostructures is solution combustion synthesis (SCS). SCS is a time and energy-saving method as compared with other routes, especially for the preparation of complex oxides which can be easily adapted for scale-up applications^44^. This method is economical both in terms of energy consumption and time. It is a simple, safe and rapid fabrication process for easily affordable porous materials due to their inherent characteristics. Many oxide nanostructures such as TiO_2_^45^, ZnO^46^, SnO_2_^47^, BiVO_4_^48^,^49^, ZrO_2_^50^, Co_3_O_4_^51^,^52^,^53^ and WO_3_^54^,^55^ have been synthesised via SCS route. Various nanocomposites including ZnO-Fe_2_O_3_^56^, TiO_2_-SiO_2_, TiO_2_-ZrO_2_, and TiO_2_-Al_2_O_3_ were also prepared by this method^57^. Therefore, we envisioned that this quick, straightforward process can be used to synthesize highly porous GO-CuFe_2_O_4_ nanocomposite that can enhance the catalytic activity of a reaction by providing more adsorption and reaction sites during the reaction.

Herein, we report the successful synthesis of highly porous GO-CuFe_2_O_4_ nanocomposite through a solution combustion route and subsequently, for the first time the use of this material as a promising heterogeneous catalyst for xanthenes synthesis have been demonstrated. The xanthenes synthesis was obtained via two-component coupling of aromatic aldehyde and 2-naphthol in the presence of GO-CuFe_2_O_4_ nanocatalyst and the same protocol was applied to other xanthene derivatives syntheses. All the reactions proceeded in a shorter period of time compared to traditional catalysts. Moreover, the catalyst can be recycled and reused up to five cycles with minimal loss in activity in a solvent free system. Our methodology may provide insight into the design of nanocatalysts using combustion route for its use in many more industrially important catalytic applications.

## Results and Discussion

### Structure and morphology characterization

Compared to the techniques employed for the preparation of nanocomposites such as solvothermal, hydrothermal and wet impregnation method, SCS is both energy and time efficient. Moreover, it can easily afford porous materials during the combustion process which is advantageous for adsorption of organic reactants on the nanocatalyst^58^. During the synthesis, hydrated nitrates as metal salts are used as metal precursors due to the efficient oxidizing power of NO_3_^−^ groups and its lower decomposition temperature and good solubility in water^59^. Urea (CO(NH_2_)_2_) was used as a common fuel due to its low cost, good availability, high exothermicity, as well as their high coordination ability toward nitrates which help in controlling the size of nanoparticles^59^. The samples obtained after the SCS synthesis was then characterized by various characterization techniques as outlined below.

**Figure 1:**
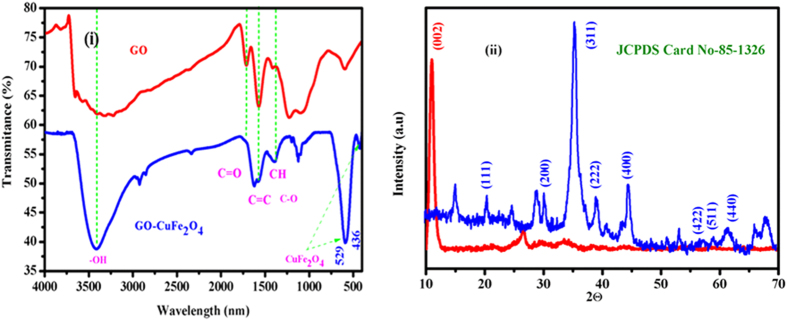
(i) FTIR spectra of GO and GO-CuFe_2_O_4_ nanocomposite and (ii) XRD powdered spectra of GO and GO-CuFe_2_O_4_ nanocomposite.

FTIR spectra were recorded to study the effective incorporation of CuFe_2_O_4_ nanoparticles on GO matrix and presence of different functional groups present in the nanocomposite material. Figure 1(i) displays FTIR spectra of the the GO-CuFe_2_O_4_ nanocomposite. As shown in Fig. 1(i), the broad absorption band at 3400–3500 cm^−1^ in the FT-IR spectra of GO sample is associated with the stretching vibration of the –OH group. The peak at 1728 cm^−1^ corresponds to the stretching of the –C=O and –COOH groups on GO sheets. The peak at 1616 cm^−1^ (aromatic C=C) can be ascribed to the skeletal vibrations of un-oxidized graphene domains. The C–O bond is associated with the band at 1047 cm^−1^. These observations are in good agreement with previous literatures^60^,^61^. In the FTIR spectrum of GO-CuFe_2_O_4_, the presence of two absorption bands at 562 cm^−1^ and 480 cm^−1^ can be noticed. The absorption band at around 529 cm^−1^ belongs to the stretching vibration of Cu^2+^ in octahedral site and the absorption band at around 436 cm^−1^ belongs to the stretching vibration of Fe^3+^ in the tetrahedral site of CuFe_2_O_4_ nanoparticles, respectively^62^. It is interesting to find that after the composite synthesis, the peaks at 1047 cm^−1^ are still present in the spectrum which suggests the minor reduction of functional groups after the composite synthesis.

XRD is a powerful technique to analyze the phase purity and crystal structure of the material. Figure 1(ii) displays the XRD spectrum of GO and GO- CuFe_2_O_4_ composites, wherein all the peaks can be assigned to cubic CuFe_2_O_4_ spinel structure. It is interesting to find that no impurity peaks of copper oxides (Cu_2_O or CuO) were observed in the spectrum^27^,^41^,^63^,^64^. A series of characteristic diffraction peaks observed at 30.5, 35.2, 57.0, 62.8 and 74.1 correspond to (220), (311), (511), (440) and (533) crystal planes of CuFe_2_O_4_, respectively (JCPDS card no: 85-1326). Moreover, no typical diffraction peak of GO (001) or RGO (002) was observed in the XRD spectrum suggesting that the GO in the GO-CuFe_2_O_4_ composite was fully exfoliated due to the crystal growth of CuFe_2_O_4_ nanoparticles between the interlayer of GO sheets, which results in the low diffraction intensity of GO^41^. The presence of broad peaks suggests the formation of smaller nanoparticles and particle size was found to be around 10 nm calculated using Debye-Scherrer formula^65^,^66^,^67^. All the above results demonstrate that during the composite synthesis GO sheets help in the controlled synthesis and the stabilization of the NPs.

**Figure 2:**
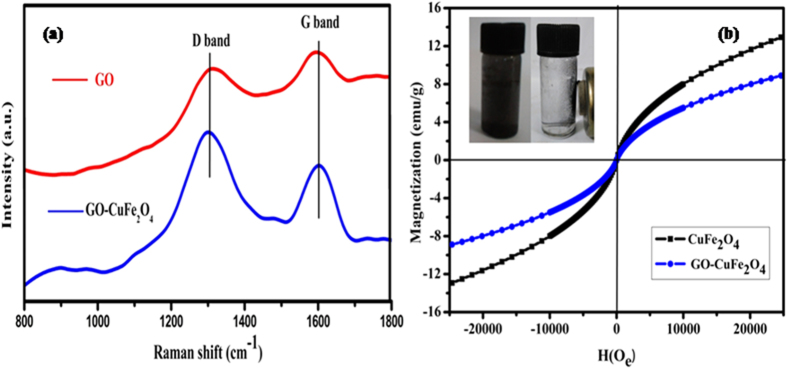
(**a**) Raman spectra of GO and GO-CuFe_2_O_4_ nanocomposite, and (**b**) Hysteresis loops of pure CuFe_2_O_4_ and GO-CuFe_2_O_4_. The inset is the magnetic separation property of GO-CuFe_2_O_4_ nanocomposite GO-CuFe_2_O_4_ nanocomposite.

To further confirm the effective reduction of GO during the composite synthesis and possible electronic interaction between GO and CuFe_2_O_4_ NPs, Raman measurements were carried out and are shown in Fig. 2(a). Similar to GO, GO-CuFe_2_O_4_ has two prominent D and G bands at 1354 and 1606 cm^−1^, respectively. A small shifting of the bands in comparison to that of GO suggest the increase in disorderness on the GO surface. It is well known that the D band arises due to the defects in the graphene sheets whereas the G band arises due to the E_2g_ mode arising due to the sp^2^ hybridised carbon domains^68^. The D/G band intensity ratio (I_D_/I_G_ = 0.97) of GO-CuFe_2_O_4_ was found to be larger than that of GO (I_D_/I_G_ = 0.86). This suggests the possible electronic interaction between the GO and CuFe_2_O_4_ NPs which results in the reestablishment of conjugated graphene oxide network (sp^2^) after loading of CuFe_2_O_4_ nanoparticles^69^.

In order to check the magnetic properties of the as-obtained pure CuFe_2_O_4_ and GO-CuFe_2_O_4_ nanocomposite, magnetic measurement was carried out by VSM technique at room temperature and the results are shown in Fig. 2(b). The magnetic hysteresis loops of CuFe_2_O_4_ and GO-CuFe_2_O_4_ are an S-like curve that confirms the strong magnetic response to a varying magnetic field. The specific saturation magnetization (Ms) was found to be 13.0 emu/g for CuFe_2_O_4_ and 8.1 emu/g for GO–CuFe_2_O_4_ composite revealing the superparamagnetic behavior of both the samples. It should be pointed out that the saturation magnetization (Ms) values decreased after the loading of GO. The values of coercivity and remanence are summarized in Table 1. When an external magnetic field was applied for 20 s, rapid aggregation of the catalysts can be observed from their homogeneous dispersion (as shown in the inset of Fig. 2b). This observation suggests that the GO-CuFe_2_O_4_ nanocatalyst can be easily separated from the solution phase using an external magnet, which is important in practical applications.

**Figure 3:**
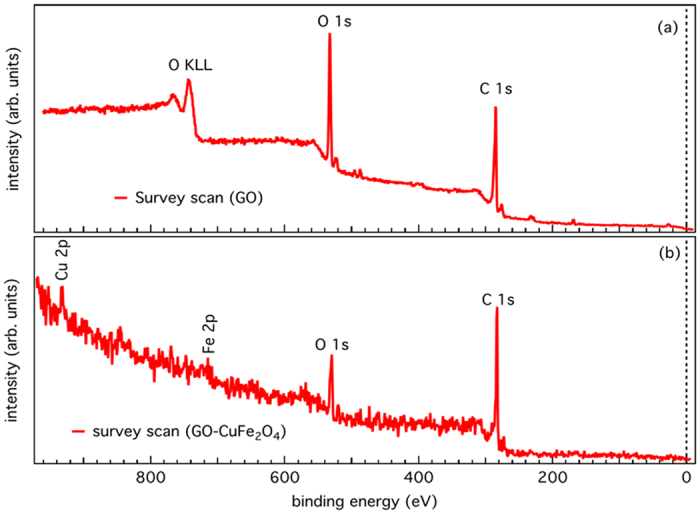
XPS survey scan of (**a**) GO and (**b**) GO-CuFe_2_O_4_ nanocomposite samples.

**Figure 4:**
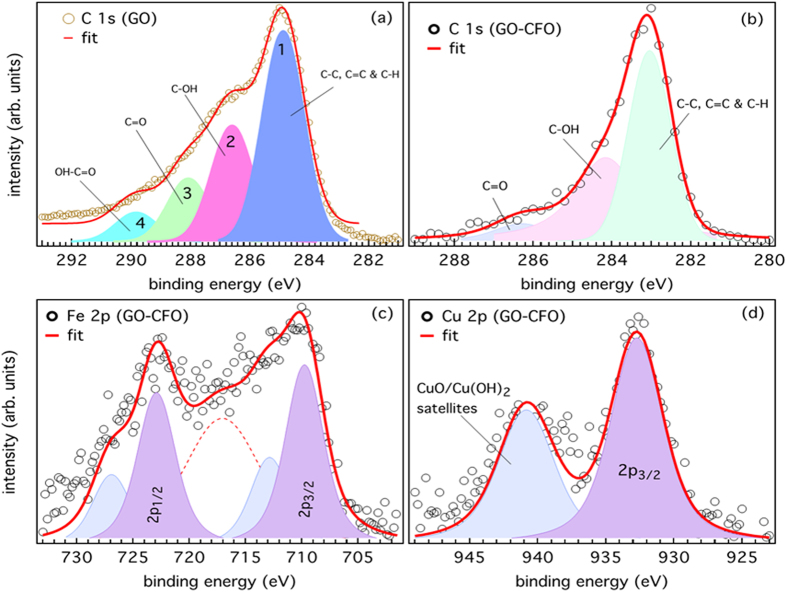
C 1s core-level spectra of (**a**) GO, (**b**) GO-CuFe_2_O_4_ nanocomposite samples. (**c**) Fe 2p and (**d**) Cu 2p core-levels of GO-CuFe_2_O_4_ sample.

A non-monochromatic Mg Kα (hv = 1253.6 eV) X-ray source and PHOIBOS 150 electron energy analyzer from SPECS GmbH, Germany was used to acquiring X-ray photoelectron spectroscopy (XPS) measurements with the base pressure below 1 × 10^−9^ mbar. We have investigated the chemical composition and electronic structure of the GO-CuFe_2_O_4_ nanocomposite. The presence of the core-level peaks in XPS survey spectra, as shown in Fig. 3(a,b), indicates the existence of Fe, Cu, O, and C elements in CuFe_2_O_4_ and GO samples. In Fig. 4(a,b), we show the C 1s XPS spectrum of GO and GO-CuFe_2_O_4_ nanocomposite samples. For GO, the main peak positioned at around 284.5 eV, is assigned to non-oxygenated ring carbon molecules, while alternate peaks at 286.7, 287.8 and 289.1 eV are assigned to the oxygen-containing groups (C–OH), (C=O), and (O=C–OH), respectively (Fig. 4a). The C 1 s core-level spectrum of GO-CuFe_2_O_4_ sample demonstrates that there is no significant change in intensity of the oxygenated functionalities in comparison to that of GO (Fig. 4b). This suggests the minor reduction of functional groups during the nanocomposite synthesis which is essential for the anchoring of metal nanoparticles on GO surface. In Fig. 4(c,d) we show the core-level spectra of Fe 2p and Cu 2p, respectively. The Fe 2p core-level spectrum shows two strong peaks at 710 and 722.8 eV, which are associated with Fe 2p_3/2_ and Fe 2p_1/2_ spin-orbit splitting, respectively. These are compared well with the Fe^3+^ octahedral species and the Fe^3+^ tetrahedral species^70^,^71^. We also observed peaks at about 3 eV below the respective Fe 2p_3/2_ (Fe 2p_1/2_) core-level peaks most probably due to FeO. Additionally, the satellite structure between the two peaks at 718 eV was the fingerprint of the electronic structure of Fe^3+^. Figure 4d shows the Cu 2p_3/2_ core level spectrum, which appears at 932.7 eV and is in accordance with Cu^2+^ states as reported in literatures^72^. A satellite peak is observed at 940.8 eV, which can be attributed to the formation of CuO. Overall, the XPS spectra of Cu 2p and Fe 2p show that Cu is in the +2 and Fe is in the +3 oxidation state in the nanocomposite, which is in good agreement with literatures on CuFe_2_O_4_ particles^73^. These results indicate successful incorporation of CuFe_2_O_4_ nanoparticles (NPs) on graphene oxide sheets.

**Figure 5:**
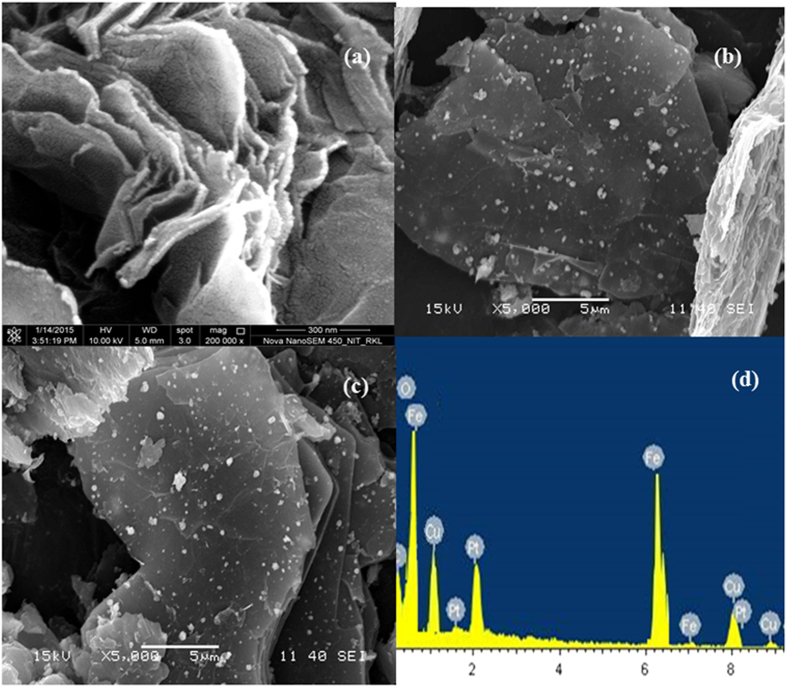
FE-SEM images of (**a**) GO and (**b,c**) GO-CuFe_2_O_4_ nanocomposite, (**d**) EDX of GO-CuFe_2_O_4_ nanocomposite.

The structural composition of GO and GO-CuFe_2_O_4_ was then ascertained by FESEM which are presented in Fig. 5. It is found from Fig. 5a that the GO exhibits thin sheet structure with wrinkled or folded morphology with a few stacked layers^74^. Furthermore, the GO nanosheets appear as an isolated lamellar structure which is convenient for magnetic CuFe_2_O_4_ particle to anchor on its surface. Unlike graphene, GO sheets are expected to be “thicker” due to the presence of carbonyl, carboxyl, hydroxyl and epoxy groups above and below the original graphene planes. Figure 5b,c shows the morphology of GO-CuFe_2_O_4_ nanocomposite. The crumpled and layered structure of GO can be easily seen in the FESEM images (5b-c). Spherical CuFe_2_O_4_ nanoparticles of larger size (~121 ± 2 nm) are found to be homogeneously distributed on the GO surface indicating the agglomeration of synthesized nanoparticles.

**Figure 6:**
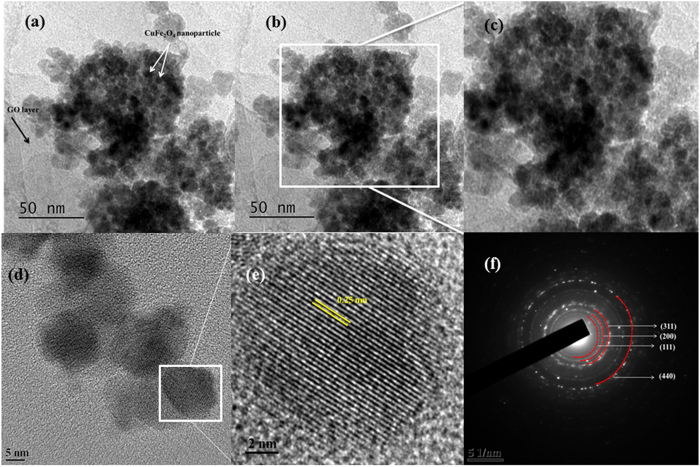
(**a–e**) TEM image of GO-CuFe_2_O_4_ nanocomposite at different magnification, (**f**) SAED image of GO-CuFe_2_O_4_ nanocomposite.

Furthermore, the energy dispersive X-ray (EDX) analyses were recorded and are shown in the Fig. 5d. Elements such as C, O, Cu and Fe can be detected in the sample of GO-CuFe_2_O_4_. From EDX analysis it was confirmed that SCS could be an excellent and efficient route for the the synthesis of graphene oxide based spinel nanocomposite. The EDX analysis showed that the distribution of the elements in the product was Cu = 14.28%, Fe = 28.95% and O = 56.77% (Table 2), thereby confirming the iron\copper ratio as 2.02 which is very much close to the atomic ratio in the formula CuFe_2_O_4_. Later on, to see the size, morphology, crystal structure, and elemental distribution in GO-CuFe_2_O_4_ composite, TEM and HRTEM with EDS mapping and scan were carried out. TEM images further demonstrate the layer structure of GO in the nanocomposite. From (Fig. 6a) TEM image, it can be seen that several individual CuFe_2_O_4_ particles seems to be agglomerated to form a bigger particle (size of 110 ± 2 nm) which sizes are found to be similar (~121 ± 2 nm) nm) to that obtained from FESEM images. The size of the individual CuFe_2_O_4_ nanoparticles is of 10 ± 2 nm which can be clearly seen from Fig. 6b. This agglomeration and interconnection of individual CuFe_2_O_4_ nanoparticles can be attributed to the powerful inherent magnetic interaction of magnetic particles^75^.

**Figure 7:**
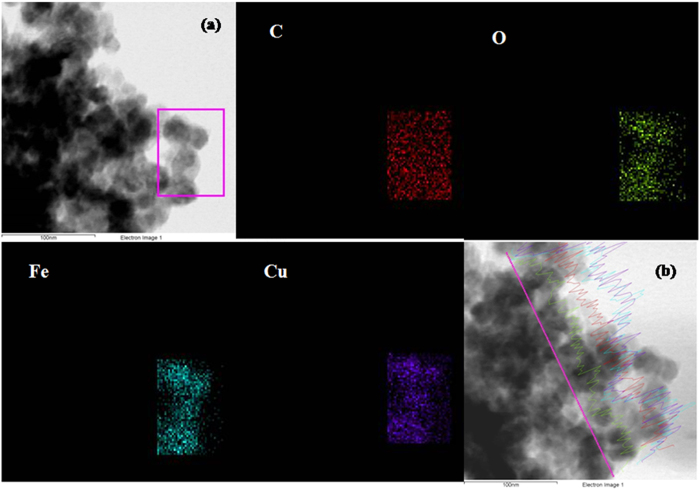
EDS mapping of (**a**) GO-CuFe_2_O_4_ nanocomposite and (**b**) EDS line scan of GO-CuFe_2_O_4_ nanocomposite.

Moreover, the NPs were not observed outside the GO sheets indicating very good interactions between NPs and GO sheets. This homogeneous distribution of the CuFe_2_O_4_ NPs can possibly be one factor for the enhanced catalytic activity as described later. The crystalline nature of the composite was further confirmed by SAED analysis which depicts a ring-like structure (Fig. 6f). The (220), (311), (400), (511) and (440) rings are indexed to the tetragonal CuFe_2_O_4_. These patterns indicated that the nanoparticles are polycrystalline. HRTEM analysis (Fig. 6d) demonstrates the crystalline structure of GO-CuFe_2_O_4_ composite. The HRTEM image of the composite shown in Fig. 6d can be resolved into lattice fringe of 0.25 nm which can be indexed to (311) plane of spinel CuFe_2_O_4_. Later on, elemental mapping was carried to determine the elemental distribution of the individual components on the surface of CuFe_2_O_4_ nanoparticles, and the results are displayed in Fig. 7a. Energy-dispersive X-ray spectrometry (EDS) mapping analysis shows that the Cu, Fe and O elements are uniformly distributed in the CuFe_2_O_4_ nanoparticles, indicating the formation of spinel nanoparticles without phase segregation, as shown in Fig. 7 (red, Cu), (green, Fe) and (blue, O). No traces of other impurities were seen in the spectra, suggesting that the synthesized composite are pure. The line scan along the direction derived in Fig. 7b demonstrated that all the elements are mixed well in the NPs. This further shows the high compositional uniformity of the GO-CuFe_2_O_4_ composite.

**Figure 8:**
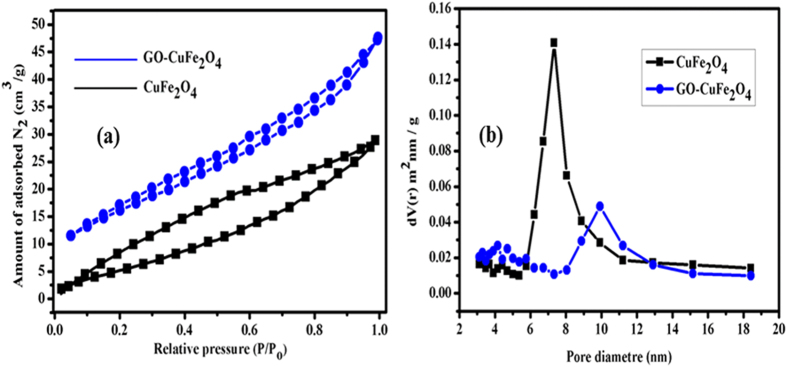
(**a**) Nitrogen adsorption/desorption isotherm and (**b**) pore size distribution of pure CuFe_2_O_4_ and GO-CuFe_2_O_4_ nanocomposite.

The surface area of the nanocomposite play an important role in the enhancement of catalytic activity by providing more adsorption and reaction sites during the reaction. To find the possible impact of SCS method on surface area and porosity, N_2_ adsorption-desorption isotherm of CuFe_2_O_4_ and GO–CuFe_2_O_4_ composite were carried out and are shown in Fig. 8a. The BET surface area of CuFe_2_O_4_ and GO-CuFe_2_O_4_ nanocomposite exhibit type IV isotherm based on the IUPAC classification. The composite had a higher specific surface area (90 m^2^·g^−1^) than that of CuFe_2_O_4_ (22 m^2^·g^−1^). The surface area is found to be higher than other reported system synthesized by solvothermal method (35 m^2^·g^−1^)^64^. The average pore diameters in GO–CuFe_2_O_4_ composite were found to be 12 nm (Fig. 8b). This observed increase in the surface area could be one of the factors responsible for the enhanced catalytic activity of the GO–CuFe_2_O_4_ composite discussed later in detail.

**Figure 9:**
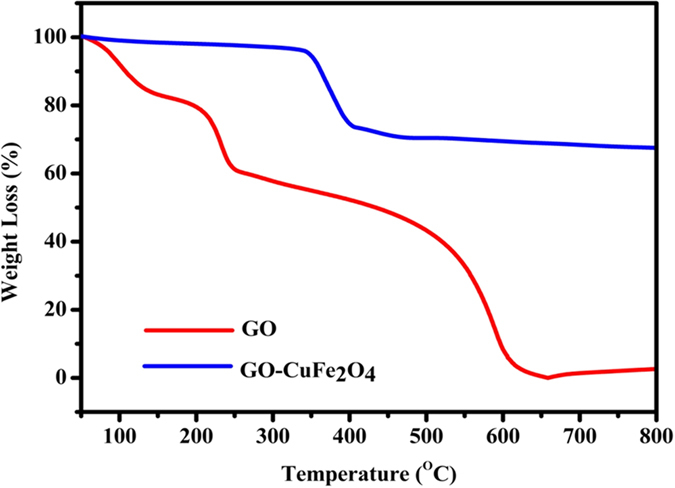
Thermogravimetric analysis (TGA) curves of GO, and GO-CuFe_2_O_4_ nanocomposite.

TGA analysis is a useful analytical tool to study thermal stability of materials and to determine the composite composition. Therefore, TGA measurements of GO and GO-CuFe_2_O_4_ composites were measured in air atmosphere and are shown in Fig. 9. The GO has two weight losses of 41.4% and 59.2% at around 200 °C and 520 °C, respectively, which can be assigned to the degradation of GO and oxidation of carbon, respectively. In comparison, GO-CuFe_2_O_4_ shows a total mass loss of 33.2% when the temperature reaches 800 °C illustrating a much higher thermal stability than GO. According to the weight loses of GO-CuFe_2_O_4_, the amount of CuFe_2_O_4_ in the GO-CuFe_2_O_4_ is estimated to be about 33.5 wt %. Later on, the actual elemental composition and percentage loading for GO-CuFe_2_O_4_ nanocomposite were further analysed by ICP-OES study. ICP analysis of 10 mg sample showed 1.96, 3.47 mg of Cu and Fe elements (i.e. 19.6% and 34.7%), respectively which means 7.43 mg of CuFe_2_O_4_ was loaded on graphene oxide sheets (i.e. 25.7% GO content). This 25.7% GO loading was in agreement with the TGA analysis as described above. ICP findings further confirmed that Cu and Fe are present in 1: 2 molar ratios which are consistent with stoichiometry of CuFe_2_O_4_ structure.

### Catalytic Reactions

Till now many acid catalyst had been reported and it was found that they are showing an efficient path for synthesis of xanthenes and its derivative. But they suffer a lot of disadvantage notably due to their prolonged reaction time, tedious work condition, use of VOCs and hazardous reaction conditions. To overcome this problem the use of ILs as solvents as well as promoter in the synthesis have been reported, but high cost of imidazole, thiazole and pyridine based IL and use of costly catalyst for the synthesis have been the essential drawbacks. To overcome this drawback use of graphene oxide based nanocomposite can be promising catalytic material. Towards this objective the reaction of 2-naphthol (2 mmol) with benzaldehyde (1 mmol) was chosen as a model reaction at 125 °C under solventless condition to assess the catalytic performance of GO-CuFe_2_O_4_ nanocatalyst. The effects of various reaction parameters on reaction of 2-naphthol with benzaldehyde were also investigated. The products were characterized by FT-IR, ^1^H-NMR and ^13^C-NMR spectroscopy.

To obtain the optimal reaction conditions, the effect of type of catalyst and catalyst dosage were initially investigated. Table 3 shows the yield of 14-phenyl-14H-dibenzo [a, j] xanthene (10a) in the presence and absence of catalyst. The reaction did not proceed in the absence of catalyst, but it increased in the presence of GO (20% yield) and CuFe_2_O_4_ nanoparticles (72% yield) (Table 3, entries 2 and 3). However, the combination of CuFe_2_O_4_ with appropriate amount of GO results in a dramatic enhancement of the catalytic activity of GO-CuFe_2_O_4_ (98% yield) (Table 3, entry 4). Since the support material takes an important role in catalysis, the impact of GO content on the catalytic activity of the GO-CuFe_2_O_4_ nanostructures was also investigated. The conversion rate of the reaction increased from 10 to 98% with increasing the loading amount of GO in GO-CuFe_2_O_4_ from 10–20% (Table 4, entries 1–5) and then decreased to 84% with further increase in loading.

This increase in catalytic activity of GO-CuFe_2_O_4_ composite can be attributed to the synergistic effect between the CuFe_2_O_4_ and the graphene oxide sheets. The presence of GO in the composite could enhance the adsorption of reactant molecules onto the catalytic sites of the GO-CuFe_2_O_4_ through π-π stacking and electrostatic interactions, leading to a high conversion rate. Additionally, the introduction of CuFe_2_O_4_ NPs into GO matrix results in its uniform dispersion on GO sheets and prevent them from agglomerating, thus increasing the number of active centers on CuFe_2_O_4_ nanoparticles. However, with further increase in GO amount beyond 20 wt% led to the lowering of active centres in the composite, which promoted less yield of the desired product. Therefore, 20 wt% was chosen as the required amount to carry out the reaction.

Later on, the effect of different dosages of GO-CuFe_2_O_4_ on xanthenes synthesis was examined (Table 5, Entry 1–7). The conversion rate increased from 60 to 98% with the rise of GO-CuFe_2_O_4_ doses from 5 to 20 mg. However, no significant difference was observed when the amount of catalyst was increased from 10 to 20 mg and yield lie above 90%. Theoretically, the amount of active sites on GO-CuFe_2_O_4_ rise with increasing dosage of catalyst, which increased the formation rate of xanthene, and would promote the reaction to reach a higher conversion rate. However, when the dosage of catalyst exceeded 20 mg, no obvious increase of the conversion rate was observed. Therefore, 20 mg of GO-CuFe_2_O_4_ was used in the subsequent experiments unless specifically stated (Table 5).

To check the efficiency of our catalyst, several other catalysts like graphite, GO, CuFe_2_O_4_,Fe_3_O_4_, CuO, Cu(NO_3_)_2,_ and Fe(NO_3_)_3_ were also compared to GO-CuFe_2_O_4_ (Table-3,entries 1–9) and it was found that GO-CuFe_2_O_4_ is the best catalyst which requires shorter reaction times with higher yield (98%). Furthermore, our catalyst showed higher activity in comparison to other GO-CuFe_2_O_4_ system synthesized by other conventional methods like solvothermal and co-precipitation methods (Tables S2). All the above results demonstrate the usefulness of our catalyst for the synthesis of xanthenes. Type of solvents has pronounced effect on a catalytic reaction. In order to study this possible impact, the reaction of 2-naphthol (2 mmol) with benzaldehyde (1 mmol) was performed in different solvents and the summary of solvent effects are presented in Table 6. It can be found that the product yield is highest when the reaction was carried out in water (92% yields, (Table 6, entry 1). When we employed dichloromethane, acetonitrile, DMSO as the solvents, product 1a was generated in slightly low yields (Table 6, entries 4, 5, 7). Meanwhile, methanol, ethanol, n-hexane, toluene and water only afforded 1a in moderate or low yields (Table 6, entries 2, 3, 8).

In addition, the reaction was carried out under solventless conditions and it was found that the reaction was carried out in very shorter reaction time and in higher yield under solventless condition (Table 6, entry 9). Therefore, solvent free conditions and using water as a solvent are obviously the best choices for these reactions. Catalytic reactions under solvent less condition are highly preferred as it reduces pollution and have low handling cost due to simplification of experimental method, workup techniques and time saving. Therefore, our method demonstrated a greener route for efficient synthesis of xanthene derivatives.

Then, we also briefly examined the effect of different temperatures, as reaction temperature has great impact on the catalysis of chemical reactions. The effect of temperatures was studied by carrying out the model reaction at different temperatures (r.t, 35, 75, 100, and 125 °C) in the presence of GO-CuFe_2_O_4_ catalyst under water and solvent less conditions (Table 7, entries 6–10). It was found that the conversion rate gradually increased with increasing of reaction temperature and the best result was obtained at 125 °C (Table 7, entries 5 and 10).

**Figure 10:**
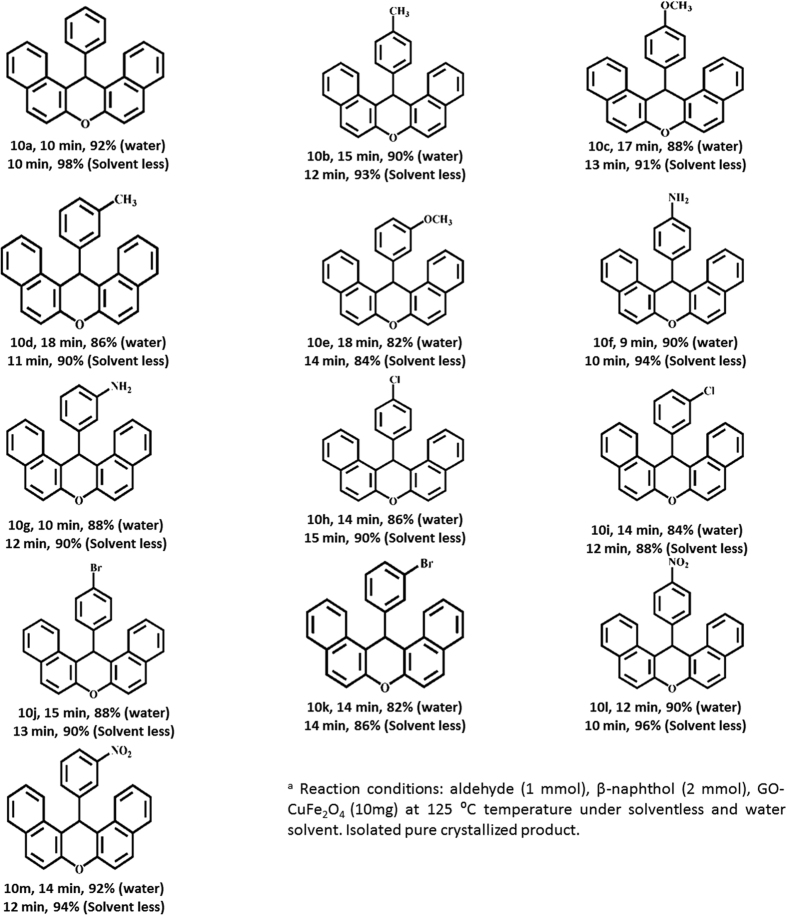
The one-pot synthesis of 14-aryl -14H-dibenzo-[a. j] xanthenes using GO-CuFe_2_O_4_ nanocomposite^a^.

After optimizing the reaction conditions, we next investigated the generality of these conditions using 2-naphthol (2 mmol) and several aldehydes (1 mmol) both in H_2_O and solvent less condition at 125 °C with 20 mg catalyst. The results are summarized in Fig. 10 (entries 10a to 10 m). It can be seen that, our composite tolerated wide range of functional groups and all the reactions were completed within 10–18 min at 125 °C to give the desired products in excellent yields (82–98%) without forming any side products. It was exciting to know that the condensation products of xanthene formation were obtained in excellent yield under solvent less condition in lesser time with respect to the systems containing H_2_O as solvent.

Later on, a wide range of aldehydes containing electron-withdrawing and electron donating groups were investigated. Aldehydes containing electron withdrawing groups such as chloro, bromo, and nitro underwent condensation in short reaction times with excellent isolated yields under both conditions (Fig. 10, entries 10 h to 10 m). Aldehyde containing electron-donating groups such as methyl, methoxy and hydroxyl required longer reaction time (Fig. 10, entries 10c to 10 g). Therefore, the presence of electron-withdrawing and electron donating groups on the phenyl ring had affected the rate of reaction which indicates that there is clear electronic role of substituent. Also, for meta and para substituted groups, the rate of the reaction was affected which explain steric effect of substituents.

The presence of acidic site on catalyst surface plays an important role in the overall catalytic activity of chemical reaction. In general, xanthene syntheses are carried out conventionally with the use of Lewis and Brönsted acid catalysts^9^,^10^,^76^. In our GO-CuFe_2_O_4_ catalyst, the acidic site in both GO and CuFe_2_O_4_ can therefore control the overall catalytic cycle. Numerous oxygen containing functionalities (e.g., alcohols and carboxylates) generated during the synthesis of GO via exhaustive oxidation provide Brönsted acidic sites to GO^77^,^78^,^79^. At the same time, CuFe_2_O_4_ has strong Lewis acid character which originates primarily from the presence of tripositive Fe^3+^ and dipositive Cu^2+^ ions. The Lewis acidity of Fe^3+^ can be attributed due to its higher electronegativity while for Cu^2+^ this is due to acquirement of a stable and completely filled d-subshell on receiving an electron^80^,^81^,^82^. As our GO-CuFe_2_O_4_ system has two acidic sites (GO as Bronsted site and CuFe_2_O_4_ as Lewis acid site) one would expect that Lewis and Brönsted acid site can play a synergistic role in the overall catalytic reaction.

In order to confirm the importance of synergistic interaction between GO and CuFe_2_O_4_, we compared the activity of GO-CuFe_2_O_4_ to that of a physical mixture of GO and CuFe_2_O_4_. It can be found that the catalytic activity of the reaction system containing physical mixture of GO and CuFe_2_O_4_ (74% yield) is similar to those observed for pure CuFe_2_O_4_ nanoparticles (72% yield), which is lower than that of GO-CuFe_2_O_4_ (98% yield) as shown in Table 3. These results indicated the possibility of synergism between GO and CuFe_2_O_4_ acidic sites in the xanthene synthesis. Similar synergistic effects in the final catalytic activity were reported in the literatures^83^,^84^,^85^,^86^. Moreover, in GO-CuFe_2_O_4_ nanocomposite which was obtained by chemical method, the CuFe_2_O_4_ nanoparticles would exhibit strong interactions with graphene oxide sheets, thereby, strongly influencing the catalytic behaviour of the nanocomposite^87^,^88^. On the other hand, a simple physical mixing cannot create effective interfacial contacts between the CuFe_2_O_4_ nanoparticles and graphene oxide sheets. As a result, slower diffusion and decrease of mass transfer of reactant molecules take place which would be the cause for the decrease in yield in case of physically mixed GO and CuFe_2_O_4_ NP system^89^.

In addition, the crucial role of CuFe_2_O_4_ nanoparticles as good catalyst was also demonstrated. As can be seen from Table 3, CuFe_2_O_4_ nanoparticles which provide smaller particle size and higher surface area showed higher activity to that of powder CuFe_2_O_4_ samples^86^,^90^. Moreover, in comparison to Fe_3_O_4_ samples, pure CuFe_2_O_4_ nanoparticles showed higher activity (table3, entry 3), demonstrating the active role of Cu^2+^ in the catalytic reaction^27^,^86^,^91^.

**Figure 11:**
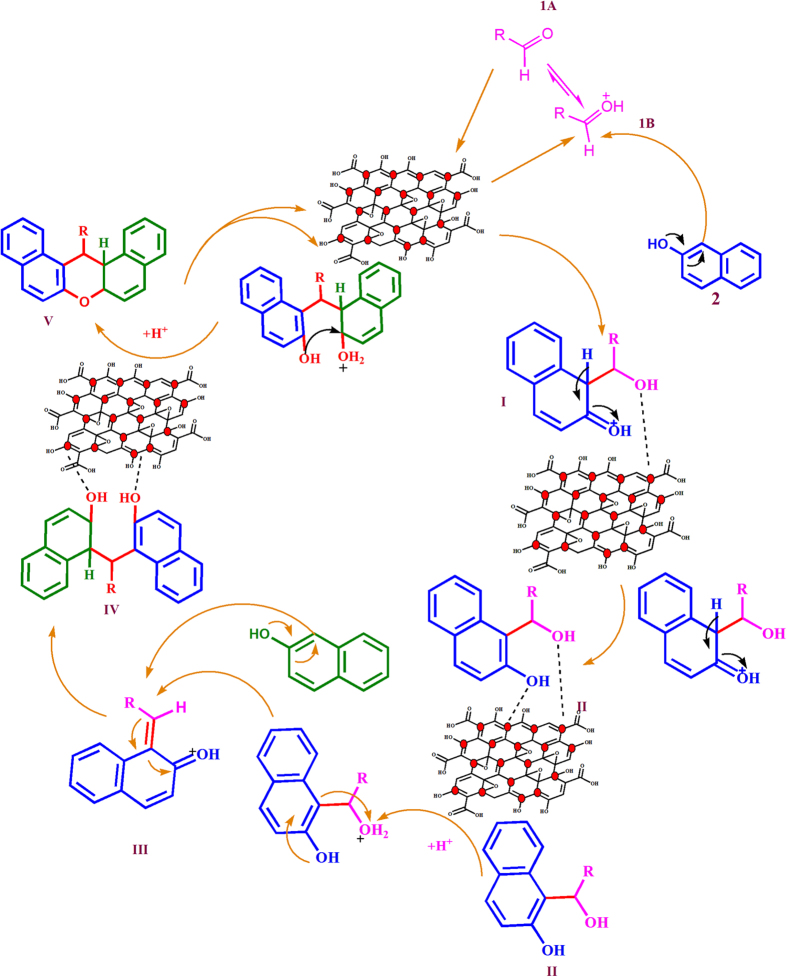
Plausible mechanism for the synthesis of xanthenes catalyzed by GO-CuFe_2_O_4_ nanocomposite.

From all the results discussed above, a plausible mechanism for xanthene synthesis using GO- CuFe_2_O_4_ catalyst has been outlined in Fig. 11. The presence of strong acidic sites (both Lewis and Brönsted acid) in GO-CuFe_2_0_4_ nanocatalyst initially facilitates the efficient chemical adsorption of reactant molecules and favours the interaction of carbonyl group of aldehyde (IA) to the acidic sites^78^,^79^,^80^. Because of this interaction, the carbonyl group of aldehyde was protonated/activated for nucleophilic attack in the subsequent steps and forms an intermediate form (IB). The lewis acid site, Cu^2+^ and Fe^3+^ of CuFe_2_O_4_ nanoparticle that anchored onto the GO surface also co-ordinate with the intermediate by bonding with the –OH group, which further increases its electrophilic nature and helps in the stabilisation of intermediate^92^,^93^. Finally, the intermediate (II) undergoes nucleophilic attack by a second molecule of β-naphthol to produce intermediate (III). The desired product is then formed by the cyclodehydration of intermediate (III) followed by dehydration. The coordination of CuFe_2_O_4_ NPs with the hydroxyl groups of intermediate help in increasing the electrophilicity of the intermediate and for subsequent nucleophilic attack of the β-naphthol molecule and in the cyclization process. It can be concluded that the Cu^2+^ and Fe^3+^ of CuFe_2_O_4_ nanoparticle help in activation and stabilization of subsrate molecules during xanthenes synthesis.

### Reproducibility of catalyst

**Figure 12:**
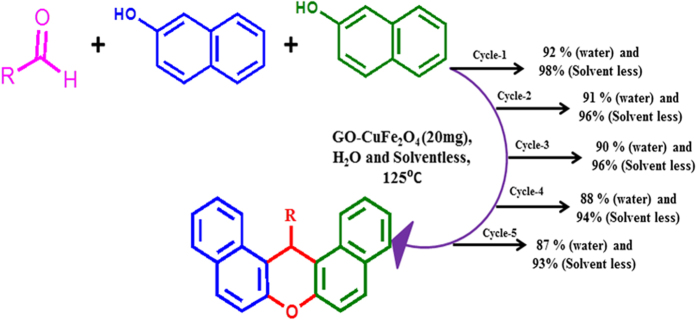
Recyclability study of the catalyst for the one-pot synthesis of 14-(phenyl) - 14H-dibenzo [a. j] xanthenes.

Recovery of catalysts is important for the re-usage and application cost of a catalyst. Since GO-CuFe_2_O_4_ composite are found to be magnetic they can be easily removed from reaction solution with a magnet. Therefore, the catalytic stability of the GO-CuFe_2_O_4_ nanocomposite for the reaction of benzaldehyde1 (1 mmol) with naphthol (2 mmol) in the presence of 20 mg of GO-CuFe_2_O_4_ nanocomposite as catalyst was studied to give the desired product. After completion of the reaction (monitored by TLC), the catalyst was separated by applying an external magnetic field, washed with deionized water and acetone, and then vacuum dried at 60 °C for 3 h before being used in the subsequent recycling experiment. As shown in Fig. 12, GO-CuFe_2_O_4_ nanocomposite was still stable even after being recycled five times. The composite showed very high activity (yield 87 and 93%) even after 5th cycle without any accountable loss of activity. The recovered catalyst was characterized by powder XRD which showed similar pattern with fresh sample indicating stability of spinel structure (see Fig. S1). Further, FESEM image (Fig. S1) showed similar morphology after 5^th^ cycle. ICP analysis also showed very minute change in percentage loading (Cu: 1.91 and Fe: 3.34) as compared to original loading 1.96, 3.47 mg of Cu and Fe elements for copper ferrite NPs. This outstanding stability and recyclability of GO-CuFe_2_O_4_ nanocomposites can be ascribed to very good interaction between CuFe_2_O_4_ NPs and GO sheets which prevents agglomeration of the nanoparticles.

### Comparison with other reported systems

Later on, to check the efficiency of our catalyst we have compared the activity of our catalyst with other reported catalysts. Table 8 shows the comparison of reported catalyst with our catalyst for the synthesis of xanthene under the same conditions. From Table 8, it can be seen that our catalyst exhibited higher yields in lesser time compared to the other reported system such as sulfamic acid^10^, [2-(sulfoxy)ethyl] sulfamic acid^94^, Fe(HSO_4_)_3_ 95, functionalized mesoporous materials^96^, InCl_3_/P_2_O_5_^97^, HY zeolite^98^ and K_5_CoW_12_O_40_.3H_2_O^99^. These results confirmed that our GO-CuFe_2_O_4_ catalyst is more effective and less time consuming for synthesis of xanthenes under solventless condition.

## Methods

Graphite powder and CDCl_3_were purchased from Sigma-Aldrich. Cu (NO_3_)_2_·3H_2_O, Fe (NO_3_)_3_·9H_2_O, H_2_O_2_ (30%), isopropanol, ethanol, NaNO_3_, H_2_SO_4_ (98%), HCl, KMnO_4_, urea and silica gel (100–200 mesh) were purchased from Hi-Media. All chemicals were used as received without further purification. 18 Milli-Ω water was used throughout the synthesis.

### Preparation of GO

GO was prepared from natural graphite powder through modified Hummers’ method^100^,^101^,^102^. In brief, 1 g of graphite was mixed to 25 ml to concentrated H_2_SO_4_ (98% w/w), followed by stirring at room temperature for 24 h. To the resulting pre-oxidized product, 100 mg of NaNO_3_ was added to the mixture and stirred for 30 min. Subsequently, the mixture was kept below 5 °C using an ice bath and 3 g of KMnO_4_ was slowly added to the mixture and the mixture was stirred for 2 h under the ice-water bath. After that about 250 ml distilled water and 20 ml H_2_O_2_ (30%) was added to dilute the solution at room temperature. After 10 min, a bright yellow solution was obtained and resulting solution was precipitated for 12 h, and the upper supernatant was collected and centrifuged. Successively, the GO powders were washed with 10% HCl and distilled water three times. The obtained GO was dispersed in distilled water to get a stable brown solution.

### Preparation of the GO-CuFe_2_O_4_ nanocatalyst

**Figure 13:**
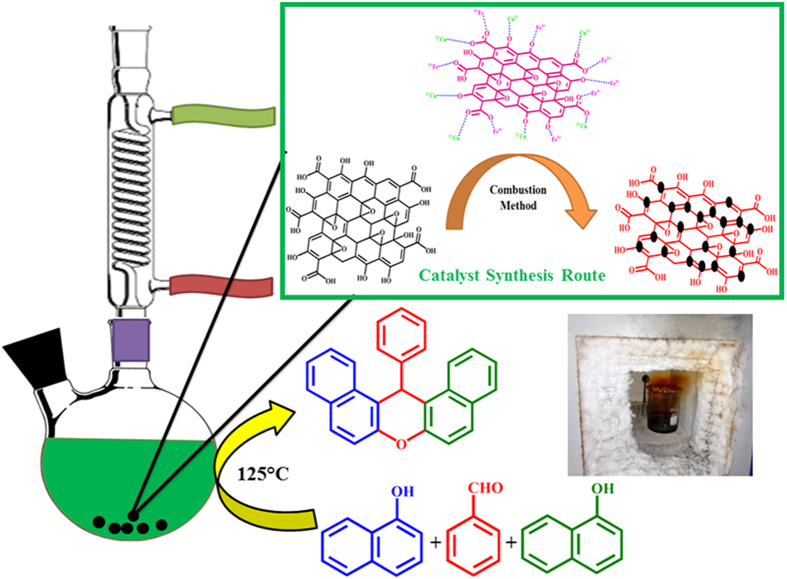
Preparation of GO-CuFe_2_O_4_ nanocomposite via SCS route for synthesis of xanthenes.

The GO–CuFe_2_O_4_ nanocomposite materials were synthesized by a solution combustion synthesis. Initially solution A was formed by adding 0.242 g (0.001 mol) sample of Cu (NO_3_)_2_·3H_2_O and 0.808 g (0.002 mol) of Fe (NO_3_)_3_· 9H_2_O to 20 mL of water and kept for stirring for 30 min at room temperature to form a homogeneous solution. Later on, solution B was prepared by dispersing 0.150 g of GO in deionized water (1.48 mL) and ethanol (18 mL), followed by 30 min ultrasonic treatment. Then, solution B was added dropwise to solution A under vigorous stirring. After 1 h stirring, urea was added as fuel to prepare the precursor solution. Finally, the precursor solution was heated on a hot plate at 100 °C to remove the solvent until a gel-like precursor was obtained. This precursor was introduced into a preheated muffle furnace at temperature of 400 °C in air and maintained for 10 min, during which combustion reaction took place. A black foam type GO–CuFe_2_O_4_ nanocomposite materials was obtained which was characterised by various analytical techniques and used as novel catalyst for xanthene synthesis (Fig. 13).

### General procedure for the synthesis of xanthenes under solventless condition

A mixture of the aldehyde (1 mmol), β-naphthol (2 mmol), and GO-CuFe_2_O_4_ (10 mg) nanocomposite was stirred at 125 °C for 10–15 min. After the completion of the reaction as monitored by TLC, the reaction mixture was cooled to room temperature and solid obtained was dissolved in EtOAc (20 mL), followed by stirring the mixture for 10 min. Later on, this was filtered to separate the catalyst. The catalyst was washed with ethyl acetate (2 × 10 mL). The combined ethyl acetate extracts were washed with water (2 × 10 mL) and dried over Na_2_SO_4_. The crude product thus obtained was washed with hexane and cold ethanol. The purified product was then obtained after column chromatography. All products were characterized by comparison of their physical constants and spectral data with those for authentic samples.

### General procedure for the synthesis of xanthenes in water media

A mixture of the aldehyde (1 mmol), β-naphthol (2 mmol), and GO-CuFe_2_O_4_ (10 mg) nanocomposite was stirred at 125 °C for 10–20 min in water as solvent. After the completion of the reaction as monitored by TLC, the reaction mixture was cooled to room temperature and solid obtained was dissolved in EtOAc (20 mL), followed by stirring the mixture for 10 min. Later on, this was filtered to separate the catalyst. The catalyst was washed with ethyl acetate (2 × 10 mL). The combined ethyl acetate extracts were washed with water (2 × 10 mL) and dried over Na_2_SO_4_. The crude product thus obtained was washed with hexane and cold ethanol. The purified product was then obtained after column chromatography. All products were characterized by comparison of their physical constants and spectral data with those for authentic samples.

### Catalyst Characterizations

The catalyst was analysed by X-ray diffraction study using PHILIPS PW 1830 X-ray diffractometer with CuKα source. The compositional information of the products was performed using EDX (JEOL JSM-6480 LV). Raman spectra were recorded using a BRUKER RFS 27 spectrometer with 1064 nm wavelength incident laser light. Field emission scanning electron microscopy (FESEM) of the sample was recorded by Nova Nano SEM/FEI. Transmission electron micrographs (TEM) of the sample were recorded using PHILIPS CM 200 equipment using carbon coated copper grids. The contents of Cu and Fe in the catalyst were analysed using ICP–OES made by Perkin Elmer, USA, ALPHA ATB. Nitrogen adsorption/desorption isotherm was obtained at 77 K on a Quanta chrome Autosorb 3-B apparatus. The specific surface area and pore size distribution were acquired by emulating BET equation and BJH method, respectively. ^1^H NMR and ^13^C NMR spectra were recorded on a Bruker spectrometer at 400 MHz using TMS as an internal standard. FTIR spectra of the product were recorded using a Perkin-Elmer FTIR spectrophotometer using NaCl support. Magnetic properties of the sample were measured by vibrating sample magnetometer (Lakeshore VSM) at room temperature. A commercial electron energy analyzer (PHOIBOS 150 from Specs GmbH, Germany) and a non-monochromatic Mg Kα x-ray source (hv = 1253.6 eV) have been used to perform XPS measurements with the base pressure of <1 × 10^−9^ mbar. Catalytic reactions were monitored by thin layer chromatography on 0.2 mm silica gel F-254 plates. All the reaction products are known compounds and have been identified by comparing their physical and spectral characteristics with the literature reported values.

## Conclusions

In conclusion, an inexpensive and magnetically recyclable GO-CuFe_2_O_4_ nanocatalyst was synthesized by a simple combustion method. Using this nanocomposite, a facile, efficient and environmentally-friendly protocol for the synthesis of xanthenes was reported. The catalyst was thoroughly characterized by a set of analytical techniques. The formation of spinel nanoparticles was comprehensively demonstrated by EDS mapping and scan techniques. We have for the first time demonstrated the use of GO-CuFe_2_O_4_ as highly active and efficient nanocatalyst for the synthesis of xanthene derivatives via one-pot two-component reaction of 2-naphthol with various aryl aldehydes under solventless condition. The results demonstrated that the combination of CuFe_2_O_4_ with graphene oxide results in a dramatic enhancement of the catalytic activity of CuFe_2_O_4_, which can be attributed to the remarkable synergistic effect between the CuFe_2_O_4_ and the graphene oxide sheets. The promising points for the presented methodology are efficiency, generality, high yield, cleaner reaction profile and recyclability which make it a useful and attractive process for the synthesis of xanthenes as biologically interesting compounds.

## Additional Information

**How to cite this article**: Kumar, A. *et al*. Greener Route for Synthesis of aryl and alkyl-14H-dibenzo [a.j] xanthenes using Graphene Oxide-Copper Ferrite Nanocomposite as a Recyclable Heterogeneous Catalyst. *Sci. Rep.* **7**, 42975; doi: 10.1038/srep42975 (2017).

**Publisher's note:** Springer Nature remains neutral with regard to jurisdictional claims in published maps and institutional affiliations.

## Supplementary Material

Supplementary Information

## Figures and Tables

**Table 1:** Magnetic properties of CuFe_2_O_4_ and GO-CuFe_2_O_4_ nanocomposite.

	Magnetic properties		
Samples	M_S_ (emu g^−1^)	H_C_ (Oe)	M_R_ (emu g^1^)
CuFe_2_O_4_	13.0	824	16.21
GO-CuFe_2_O_4_	8.1	1246	8.46

**Table 2:** EDX compositions of GO-CuFe_2_O_4_ nanocomposite.

Element	Weight %	Atomic %	Atomic ratio
O	56.77%,	82.6	
Fe	28.95%	11.8	2.02
Cu	14.28%	5.6	1

**Table 3:** Effect of different catalysts on the reaction of 2-naphthol and benzaldehyde^a^.

Entry	Catalyst	Yield (%)^b^
1.	Graphite	10
2.	GO	20
3.	CuFe_2_O_4_ nanoparticle	72
4.	GO/CuFe_2_O_4_	98
5.	CuO	52
6.	Fe_3_O_4_	40
7.	Cu(NO_3_)_2_	12
8.	Fe(NO_3_)_3_	4
9.	Without catalyst	traces
10.	GO + CuFe_2_O_4_ physical mixing	74
11.	CuFe_2_O_4_ powder	52

^a^Reaction conditions: 2-naphthol (2 mmol) and benzaldehyde (1 mmol). ^b^Isolated yield.

**Table 4:** Effect of different amount of catalysts loading. on the reaction of 2-naphthol and benzaldehyde^a^.

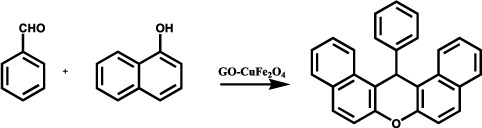			
Entry	Catalyst	Catalyst loading	Yield^b^ (%)
1.	GO-CuFe_2_O_4_	5 wt %	70
2.	GO-CuFe_2_O_4_	10 wt %	82
3.	GO-CuFe_2_O_4_	20 wt %	98
4.	GO-CuFe_2_O_4_	30 wt %	92
5.	GO-CuFe_2_O_4_	40 wt %	84

^a^Reaction conditions: 2-naphthol (2 mmol) and benzaldehyde (1 mmol). ^b^Isolated yield.

**Table 5:** Synthesis of 14H-dibenzo [a. j] xanthene using different amount of catalysts^a^.

Entry	Amount of catalyst (mg)	Time (min)/% yield^b^
1.	Nil	60/nil
2.	5	30/60
3.	10	20/90
4.	15	15/92
5.	20	10/98

^a^Reaction conditions: 2-naphthol (2 mmol) and benzaldehyde (1 mmol). ^b^Isolated yield.

**Table 6:** Effect of solvent on the condensation of 14H-dibenzo [a. j] xanthene using GO-CuFe_2_O_4_ nanocatalyst^a^.

Entry	Solvent	Catalyst	Catalyst loading	Temp (°C)	Yield^b^ (%)
1.	H_2_O	GO-CuFe_2_O_4_	20 wt %	125	92
2.	Methanol	GO-CuFe_2_O_4_	20 wt %	125	88
3.	Ethanol	GO-CuFe_2_O_4_	20 wt %	125	84
4.	DCM	GO-CuFe_2_O_4_	20 wt %	125	42
5.	Acetonitrile	GO-CuFe_2_O_4_	20 wt %	125	50
6.	Toluene	GO-CuFe_2_O_4_	20 wt %	125	60
7.	DMSO	GO-CuFe_2_O_4_	20 wt %	125	68
8.	Hexane	GO-CuFe_2_O_4_	20 wt %	125	20
9.	Solvent less	GO-CuFe_2_O_4_	20 wt %	125	98

^a^Reaction conditions: 2-naphthol (2 mmol) and benzaldehyde (1 mmol). ^b^Isolated yield.

**Table 7:** Effect of temperature on the condensation of 14 H-dibenzo [a. j] xanthene using GO-CuFe_2_O_4_ nanocatalyst^a^.

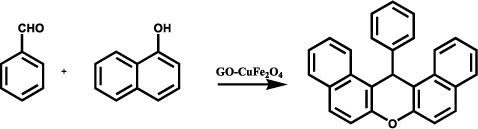					
Entry	Solvent	Catalyst	Catalyst loading	Temp (°C)	Yield^b^ (%)
1.	Water	GO-CuFe_2_O_4_	20 wt % GO/CuFe_2_O_4_	r.t	8
2.	Water	GO-CuFe_2_O_4_	20wt % GO/CuFe_2_O_4_	35	30
3.	Water	GO-CuFe_2_O_4_	20wt % GO/CuFe_2_O_4_	70	58
4.	Water	GO-CuFe_2_O_4_	20 wt % GO/CuFe_2_O_4_	100	78
5.	Water	GO-CuFe_2_O_4_	20 wt % GO/CuFe_2_O_4_	125	92
6.	Solventless	GO-CuFe_2_O_4_	20 wt % GO/CuFe_2_O_4_	r.t	10
7.	Solventless	GO-CuFe_2_O_4_	20 wt % GO/CuFe_2_O_4_	35	40
8.	Solventless	GO-CuFe_2_O_4_	20 wt % GO/CuFe_2_O_4_	70	75
9.	Solventless	GO-CuFe_2_O_4_	20 wt % GO/CuFe_2_O_4_	100	88
10.	Solventless	GO-CuFe_2_O_4_	20 wt % GO/CuFe_2_O_4_	125	98

^a^Reaction conditions: 2-naphthol (2 mmol) and benzaldehyde (1 mmol). ^b^Isolated yield.

**Table 8:** Comparison of the catalytic activity of GO-CuFe_2_O_4_ nanocomposite with the reported catalysts in the preparation of 14-(phenyl)-14H dibenzo [a. j] xanthenes.

Entry	Catalyst	Amount of catalyst (mol %)	Temperature (°C)	Time (min)	Yield (%)	Solvent	Ref.
1.	Sulfamic acid	10	125	660	94	Solventless	10
2.	[2-(Sulfoxy)ethyl]sulfamic acid	15	150	20	76		94
3.	GO-CuFe_2_O_4_	10	125	10	98	Solventless	Present Work
4.	GO-CuFe_2_O_4_	10	125	15	92	H_2_O	Present Work
5.	Fe(HSO_4_)_3_	35	—	420	85	DCM	95
6.	Functionalized mesoporous materials	20	25	360	80	DCM	96
7.	InCl_3_/P_2_O_5_	—	120	25–80	58–88		97
8.	HY zeolite	—	80	60–1440	80–94	Solventless	98
9.	K_5_CoW_12_O_40_.3H_2_O	—	125	120	91	Solventless	99
