# Supplementary material for: Greener Route for Synthesis of aryl and alkyl-14H-dibenzo [a.j] xanthenes using Graphene Oxide-Copper Ferrite Nanocomposite as a Recyclable Heterogeneous Catalyst
Source: Sci Rep. 2017 Feb 24;7:42975. doi: 10.1038/srep42975 (PMC5324042; doi:10.1038/srep42975)
Supplement: Supplementary Information [file srep42975-s1.pdf]

## Supporting Information

# Greener Route for Synthesis of aryl and alkyl-14H-dibenzo [a.j] xanthenes using Graphene Oxide-Copper Ferrite Nanocomposite as a Recyclable Heterogeneous Catalyst

Aniket Kumar,[a] Lipeeka Rout,[a] Lakkoji Satish Kumar Achary,[a] Rajendra. S. Dhaka[b] and Priyabrat Dash*[a]

[a] Department of Chemistry, National Institute of Technology, Rourkela, Odisha, India, 769008
[b] Novel Materials and Interface Physics Laboratory, Department of Physics, Indian Institute of Technology Delhi, Hauz Khas, New Delhi-110016, India.

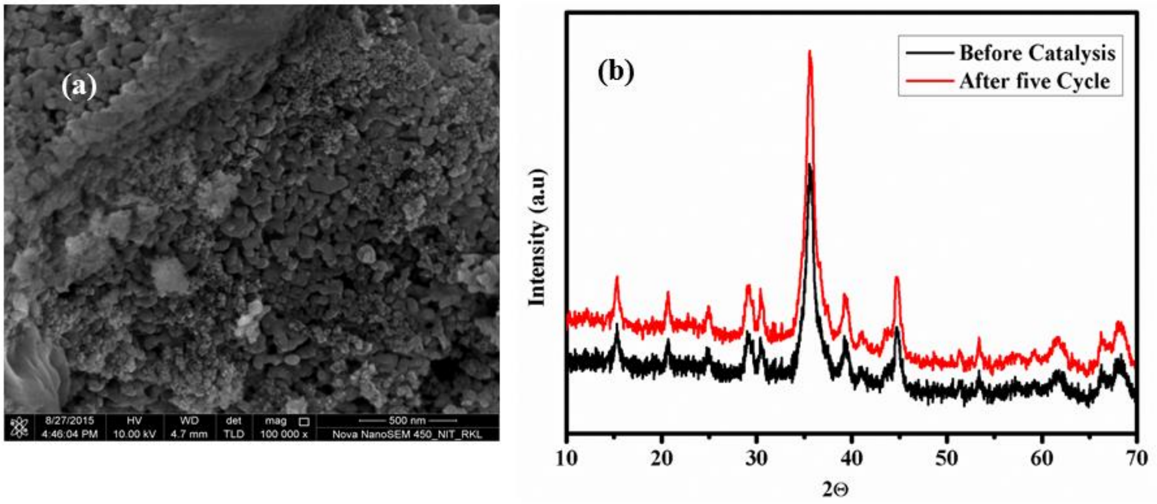


**S1** (a) FESEM and (b) powder XRD spectrum of magnetically recovered GO-$CuFe_2O_4$ after 5th catalytic run

**S2.** Comparison table for catalysis using different method for nanocomposite synthesis.

| SR.NO | Method | Yield/Time |
|---|---|---|
| 1 | Combustion | 98/10 |
| 2 | Solvothermal | 23/10 |
| 3 | Co-Precipitation | 37/10 |

**S3. Spectral analysis data of some selected compound**

Selected spectroscopic data ($^1$H and $^{13}$C NMR) of xanthene derivative

**Figure 10, entry 1(10a); 14-phenyl-14H-Dibenzo [a, j] xanthene**

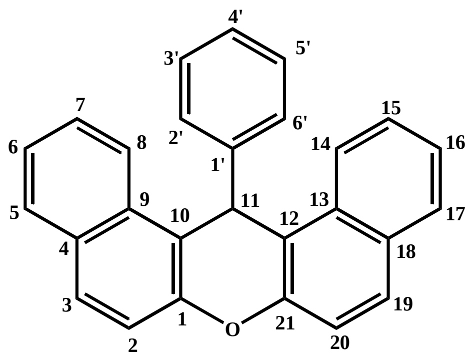


Melting point: 182-183°C

$^1$H NMR (400 MHz, $CDCl_3$): δ (ppm) 6.44 (1H, s, 11-CH), 7.24 (2H, s, Ar–H), 7.37–7.50 (9H, m), 7.74–7.79 (4H, m), 8.33–8.36 (2H, d, Ar–H).

$^{13}$C NMR (100 MHz, $CDCl_3$): δ (ppm) 38.10, 117.39, 118.05, 122.73, 124.22, 126.77, 128.26, 128.54, 128.85, 131.14, 131.58, 145.02, 148.80

**Figure 10, entry 1(10b); 14-(4-methylphenyl-14H-Dibenzo [a, j] xanthene**

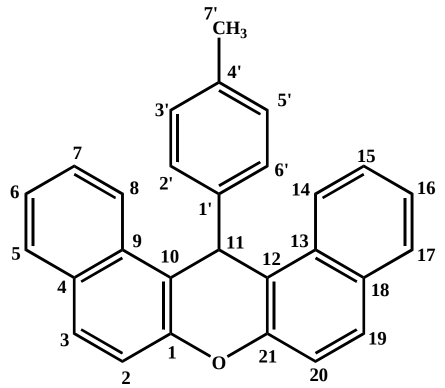


Melting point: 226-227°C

$^1$H NMR (400 MHz, $CDCl_3$): δ (ppm) 5.286 (1H, s, 11-CH), 8.031–7.068 (11H, m, ArH), 2.579 (2H, s, $CH_2$), 2.353–2.225 (2H, m, $CH_2$), 1.126 (3H, s, $CH_3$), 0.973 (3H, s, $CH_3$).

$^{13}C$ NMR (100 MHz, $CDCl_3$): δ(ppm) 196.84, 163.81, 147.59, 144.62, 131.35, 131.26, 128.71, 128.30, 128.11, 126.88, 126.12, 124.77, 124.14, 123.54, 117.56, 116.92, 114.12, 50.75, 41.26, 34.57, 32.14, 29.18, 27.02.

**Figure 10, entry 1(10c); 14-(4-methoxyphenyl-14H-Dibenzo [a, j] xanthene**

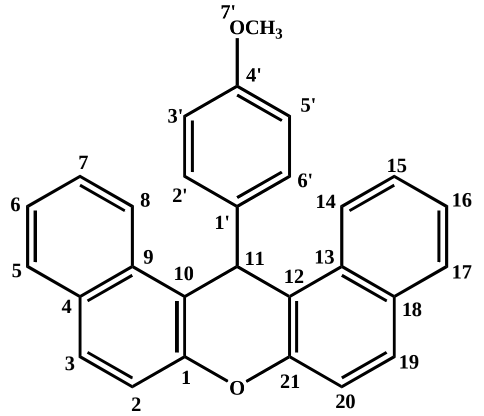


Melting point: 204-205°C

$^{1}H$ NMR (400 MHz, $CDCl_3$): δ (ppm) 6.481 (1H, s, 11-CH), 8.434 (2H, d, ArH), 8.089–7.417 (12H, m, ArH), 6.997 (2H, d, ArH), 2.196 (3H, s, $CH_3$).
$^{13}C$ NMR (100 MHz, $CDCl_3$): δ (ppm) 148.66, 142.15, 135.90, 131.46, 131.08, 129.19, 128.79, 128.12, 126.76, 124.22, 122.73, 118.01, 117.45, 37.64, 20.91.

**Figure 10, entry 1(10h); 14-(4-chlorophenyl-14H-Dibenzo [a, j] xanthene**

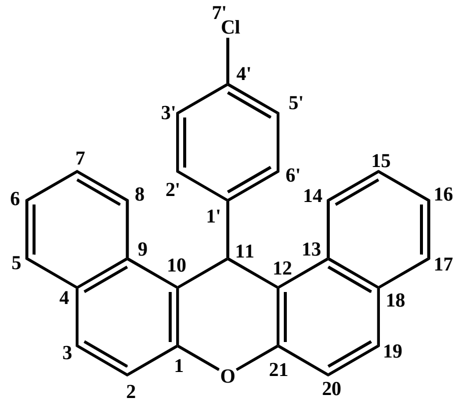


Melting point: 288-289°C

$^{1}H$ NMR (400 MHz, $CDCl_3$): δ (ppm) 6.579 (1H, s, 11-CH), 8.457 (2H, d, ArH), 7.905–7.844 (4H, m, ArH), 7.664–7.450 (8H, m, ArH), 7.155 (2H, d, ArH).

$^{13}C$ NMR (100 MHz, $CDCl_3$): δ (ppm) 147.22, 142.73, 130.52, 129.86, 129.69, 128.47, 127.95, 127.64, 127.31, 125.88, 123.34, 121.53, 116.77, 115.51, 35.76.

**Figure 10, entry 1(10i); 14-(3-chlorophenyl-14H-Dibenzo [a, j] xanthene**

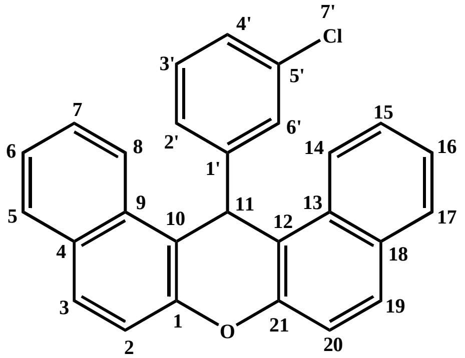


Melting point: 262-263°C

$^{1}H$ NMR (400 MHz, $CDCl_3$): δ (ppm) 6.36 (1H, s, 11-CH), 6.85–6.88 (1H, d), 6.95–6.99 (1H, t), 7.30–7.40 (6H, m), 7.47–7.51 (2H, t), 7.69–7.71 (4H, m), 8.21–8.23 (2H, d, Ar–H).

$^{13}C$ NMR (100 MHz, $CDCl_3$): δ (ppm) 37.75, 116.58, 118.08, 122.40, 124.38, 126.40, 126.74, 126.96, 128.33, 128.90, 129.16, 129.60, 131.06, 131.27, 134.42, 146.88, 148.79.

**Figure 10, entry 1(10j); 14-(4-bromophenyl-14H-Dibenzo [a, j] xanthene**

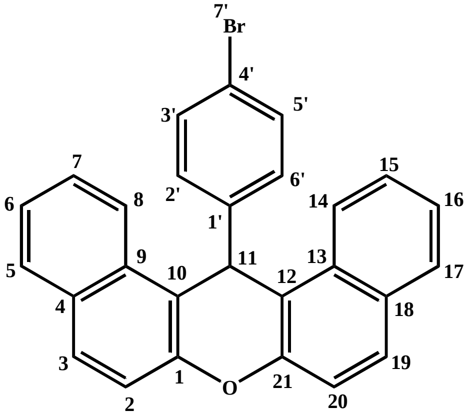


Melting point: 294-295°C

$^{1}H$ NMR (400 MHz, $CDCl_3$): δ (ppm) 5.21 (1H, s, 11-CH), 7.16–7.18 (2H, d, Ar–H), 7.29–7.40 (6H, m), 7.48–7.52 (2H, t, Ar–H), 7.71–7.76 (4H, m), 8.22–8.24 (2H, d, Ar–H).

$^{13}C$ NMR (100 MHz, $CDCl_3$): δ (ppm) 37.72, 115.89, 118.12, 121.69, 122.02, 122.71, 124.58, 127.24, 129.06, 129.49, 129.56, 131.04, 131.06, 134.2.

**Figure 10, entry 1(10k); 14-(3-bromophenyl-14H-Dibenzo [a, j] xanthene**

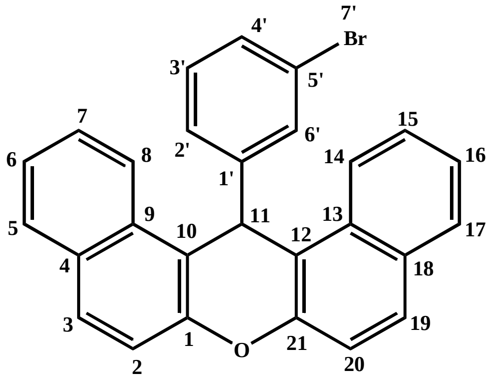


Melting point: 293-294°C

$^{1}H$ NMR (400 MHz, $CDCl_3$): δ (ppm) 6.59 (s, 11-CH),8.51 (d, 1H), 7.85-7.80 (m, 4H), 7.52 (t, 2H), 7.47-7.34 (m, 5H), 7.21 (d, 2H), 7.10-6.90 (m, 2H).

$^{13}C$ NMR (100 MHz, $CDCl_3$): δ (ppm) 149.3, 143.2, 132.5, 131.0, 130.9, 130.2, 130.1, 129.2, 129.0, 128.7, 128.5, 126.3, 125.0, 123.2, 119.2, 116.3, 35.6

**Figure 10, entry 1(10l); 14-(4-nitrophenyl-14H-Dibenzo [a, j] xanthene**

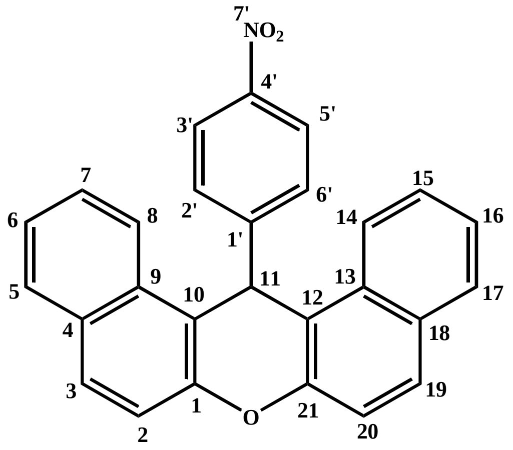


Melting point: 312-313°C

$^{1}H$ NMR (400 MHz, $CDCl_3$): δ (ppm) 6.612 (1H, s, 11-CH), 8.291 (2H, d, ArH), 8.008 (2H, d, ArH), 7.874–7.831 (4H, m, ArH), 7.700–7.421 (8H, m, ArH),

$^{13}C$ NMR (100 MHz, $CDCl_3$): δ (ppm) 148.72, 131.03, 129.55, 129.02, 128.92, 127.15, 124.54, 123.83, 121.98, 118.02, 115.71, 37.82.

**Figure 10, entry 1(10m); 14-(3-nitrophenyl-14H-Dibenzo [a, j] xanthene**

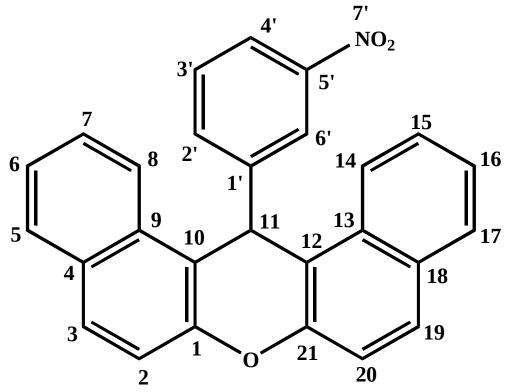


Melting point: 210-212°C

$^{1}H$ NMR (400 MHz, $CDCl_3$): δ (ppm) 6.60 (1H, *s*, 11-CH), 6.65 (1H, *s*, Ar-H) 7.41–7.44 (3H, *m*, Ar-H), 7.48 (2H, *d*, Ar-H), 7.51–7.54 (4H, *m*, Ar-H), 7.64 (2H, *d*, Ar-H), 7.83–7.87 (4H, *m*, Ar-H);

$^{13}C$ NMR (100 MHz, $CDCl_3$): δ (ppm) 37.3 (CH), 116.3, 116.8, 117.4, 121.1, 122.3, 125 136.3, 137.1, 138.2, 146.5, 147.9, 158.3.
